# Supplementary material for: Immunodynamics of explanted human tumors for immuno‐oncology
Source: EMBO Mol Med. 2020 Dec 29;13(1):e12850. doi: 10.15252/emmm.202012850 (PMC7799366; doi:10.15252/emmm.202012850)
Supplement: Supplementary file 1 — Appendix [file EMMM-13-e12850-s001.pdf]

Immunodynamics of Explanted Human Tumors for Precision and Personalized Immuno-Oncology.

APPENDIX

Table of content

Appendix Figure S1. Flow cytometry (D0) gating strategy
Appendix Figure S2. Cancer immune atlas of tumors based on flow cytometry analyses at baseline
Appendix Figure S3. *In sitro* incubation with isotype control mAb (IgG4) was not statistically different from medium
Appendix Figure S4. Anergic tumor infiltrates and correlation between targets expressions and response to combinatorial regimen
Appendix Table S1. Patient's clinical characteristics at surgery and sample use
Appendix Table S2. List of each tumor sample indicating weight (when available) and protein content by Bradford assay
Appendix Table S3. Consort flow chart of the different assays performed on tumor samples
Appendix Table S4. Immune reactivity score (IRS) after anti-PD-1 mAbs stimulation based on Figure 1d algorithm
Appendix Table S5. Correspondance between IRS and clinical outcome
Appendix Table S6. Clinical characteristics of the lung cancer patients cohorts for the prognostic value of tumoral CXCL10 mRNA
Appendix Table S7. Contingency IL-2/anti-PD-1 responders (R) *versus* non-responders (NR)
Appendix Table S8. Scoring of hypo-responsiveness for each tumor sample based on figure 4d and reversion by anti-KIR + anti-PD-1

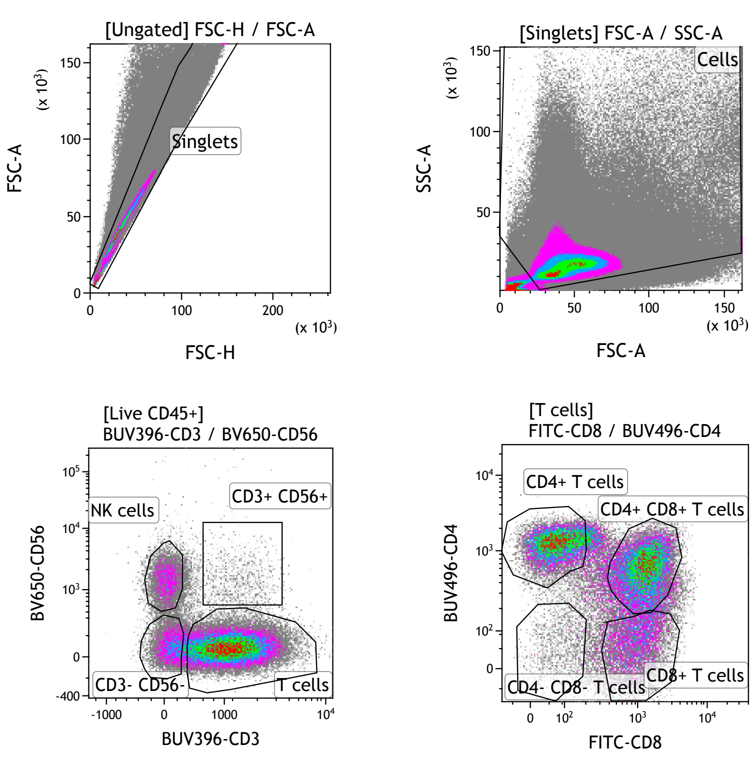

Appendix **Figure S1. Flow cytometry (D0) gating strategy.** Representative example of the gating strategy used to evaluate CD45- and CD45+ cells and corresponding cell subsets.

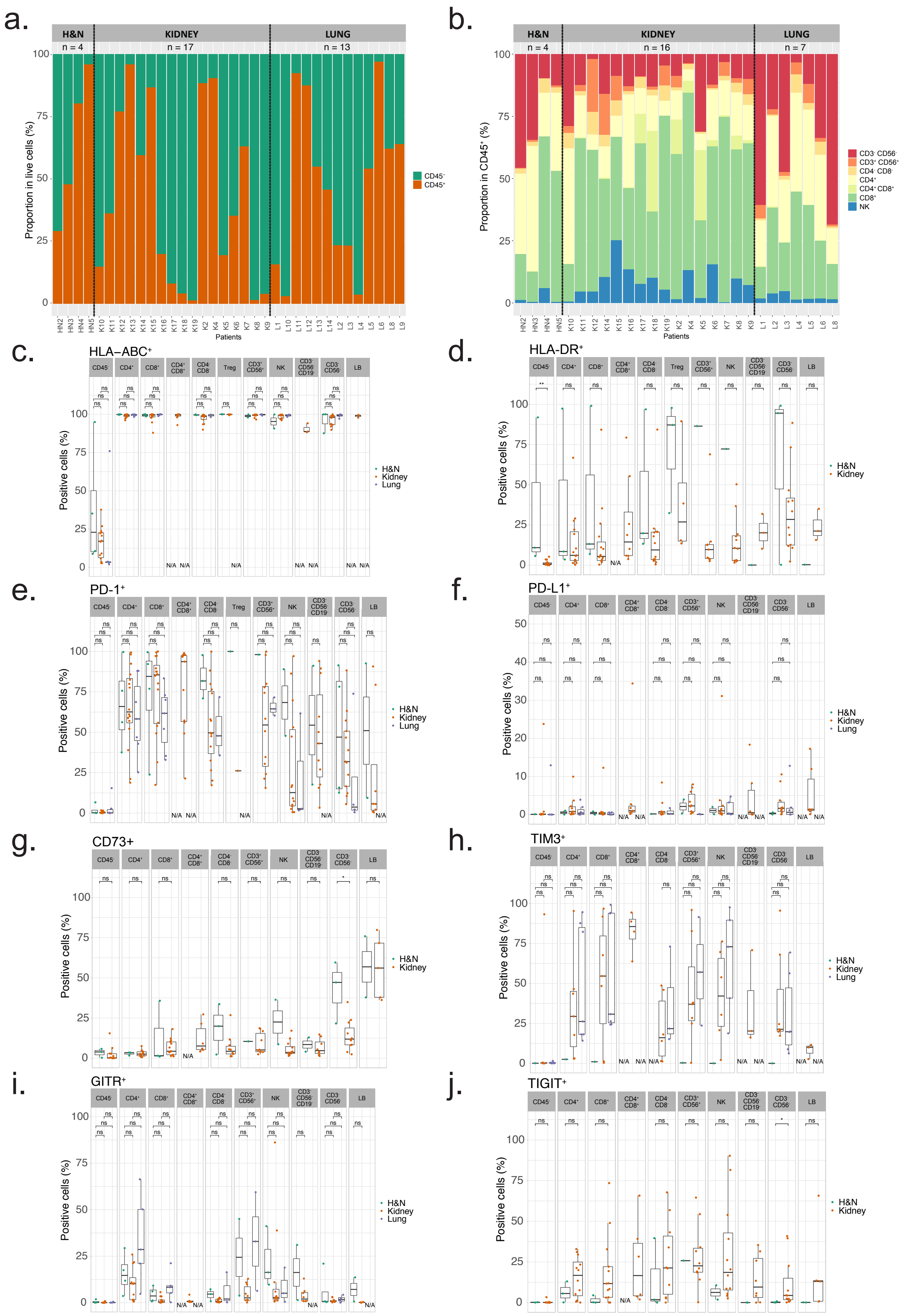

Appendix **Figure S2. Cancer immune atlas of tumors based on flow cytometry analyses at baseline. a-b.** Bar plots showing the fractions of CD45$^+$ and CD45$^-$ cells in live cells (a, *n*=34) and various subsets of leucocytes (b, *n*=27) in CD45$^+$ cells by tumor type (head and neck (HN), kidney (K) and lung (L)) available for analyses. **c- j.** Dot plots showing cell surface expression of various activation or exhaustion markers of CD45$^-$ cells or amongst

CD45+ cells of each indicated cell subtype by tumor type. Flow cytometry-based percentages of expression of HLA-ABC (c), HLA-DR (d), PD-1 (e), PD-L1 (f), CD73 (g), TIM3 (h), GITR (i) and TIGIT (j). Each dot represents one tumor. Statistical analyses: Wilcoxon rank-sum tests. A Benjamini–Hochberg (BH) correction procedure was performed when more than 10 statistical tests were performed. Only data ≥ 500 events were plotted here. N/A: data not available or < 500 events.

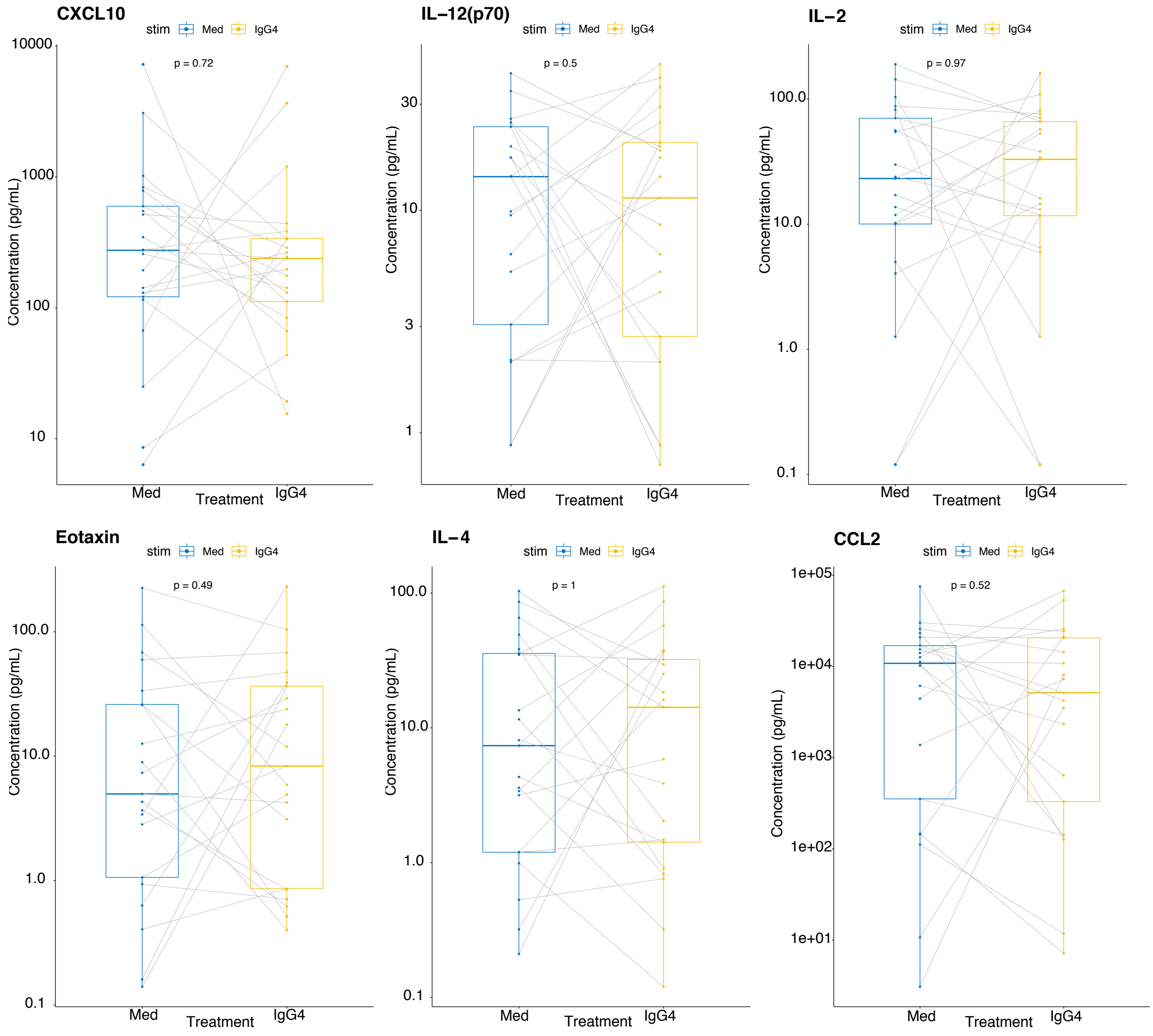

Appendix **Figure S3. *In sitro* incubation with isotype control mAb (IgG4) was not statistically different from medium.** The immune-reactivity to anti-PD-1 mAbs (IgG4) of the tumor immune infiltrates were compared to isotype control IgG4 antibody or medium alone. The 6 SFs displayed are representative of the results obtained with the 27 SFs. Values for representative SFs are depicted for each tumor. Wilcoxon rank-sum paired test: ns=not significant (p>0.05).

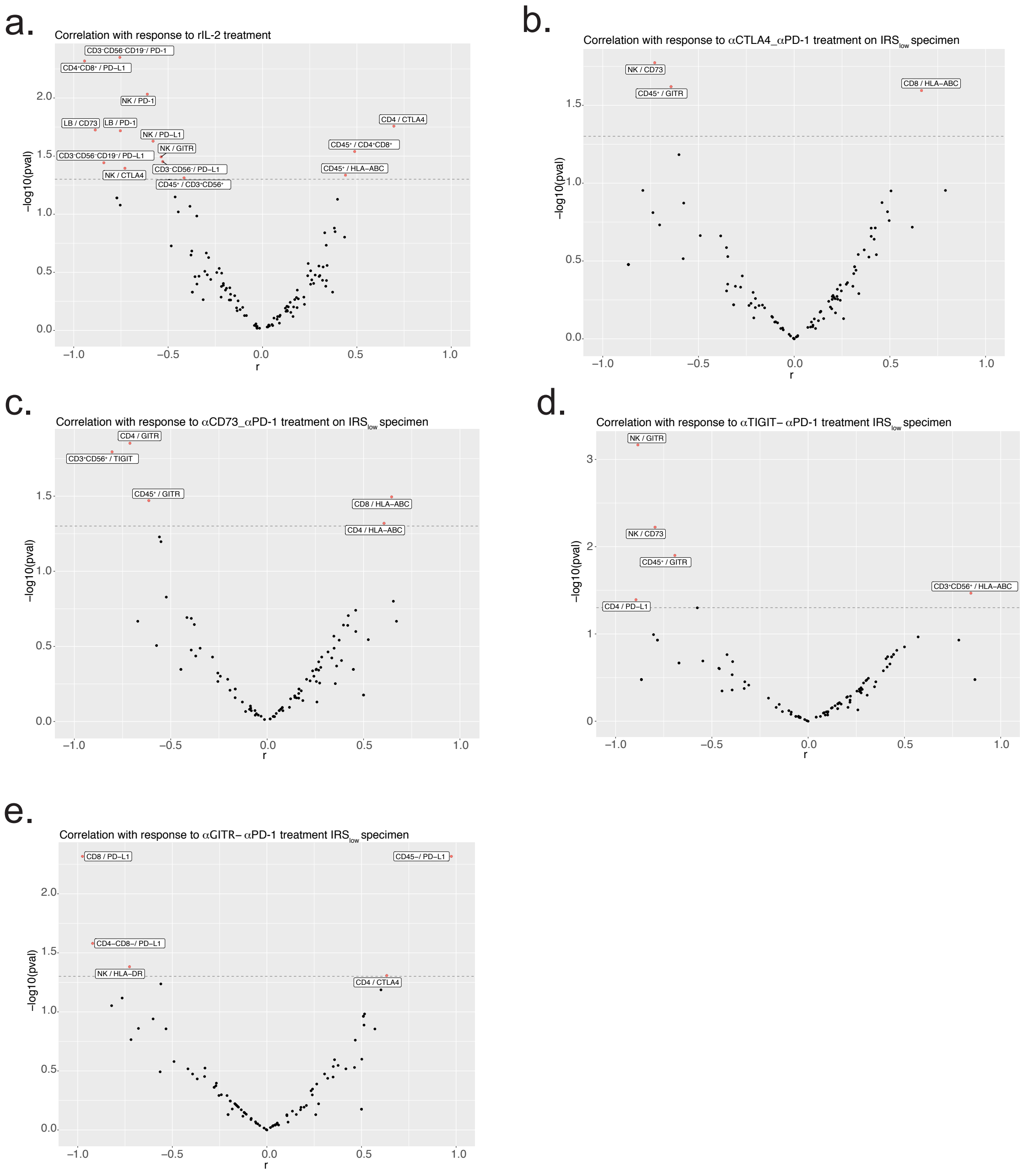

Appendix **Figure S4. Anergic tumor infiltrates and correlation between targets expressions and response to combinatorial regimen.**

a. Volcano plot of the responses after rIL-2 stimulation and the phenotype of TILs at baseline. Log10-transformed Wilcoxon rank-sum test p-values and the log10-transformed ratio

according to the immune reactivity score after rIL-2 blockade (fold ratio *versus* medium). Each dot represents one population; associated biomarkers are highlighted and filled in red ($p<0.05$). rIL-2 resistance (anergy) is correlated with PD-1 expression on various immune cell subtypes explaining the possibility to reverse anergy with PD-1 blockade treatments (see also Appendix Table S7). **b-e.** Representative volcano plots of combinatorial regimen and relative expression of their target molecule at Day 0. Expression of targets for anti-CTLA4 + anti-PD-1 (b), anti-CD73 + anti-PD-1 (c), anti-TIGIT + anti-PD-1 (d) or anti-GITR + anti-PD-1 (e) do not correlate with response to combination treatments. Statistical analyses: Wilcoxon rank-sum test, p.value < 0.05.

Appendix Table S1. Patients' clinical characteristics at surgery and sample use

Variables		Renal cell carcinomas (N=20)	Non–small cell lung carcinomas (N=16)	Bladder carcinomas (N=2)	Head and Neck carcinomas (N=4)	Ovarian carcinoma (N=1)
Gender n (%)	Female	5 (25%)	4 (25%)	1 (50%)	1 (25%)	1 (100%)
	Male	12 (60%)	12 (75%)	1 (50%)	3 (75%)	–
	Missing	3 (15%)	4 (25%)	–	–	–
Age (years)	Median	64	67	–	59	59
	Range	45–86	35–78	–	46–79	–
Smoking status n (%)	Smoker	–	4 (25%)	–	2 (50%)	–
	Nonsmoker	–	0 (0%)	–	2 (50%)	–
	Missing	–	12 (75%)	–		–
Pathological type n (%)	Squamous cell cacinoma	–	4 (25%)	1 (50%)	3 (75%)	–
	Non Squamous cell cacinoma	–	11 (69%)	–	–	–
	Clear cell	13 (65%)	–	–	–	–
	Papillary	4 (20%)	–	–	–	–
	Urothelial carcinoma	–	–	1 (50%)	–	–
	Other	–	–	–	1 (25%)	1 (100%)
	N/A	3 (15%)	1 (6%)	–	–	–
Disease stage n (%)	Localized	13 (65%)	9 (56%)	2 (100%)	3 (75%)	1 (100%)
	Advanced IV	4 (20%)	2 (13%)	–	1 (25%)	–
	N/A	3 (15%)	5(31%)	–	–	–
Neoadjuvant n (%)	Chemotherapy	–	–	–	–	1 (100%)
	Immunotherapy	–	1 (6%)	–	–	–
Immunotherapy (ICB) after *in sitro* assay	n (%)	3 (20%)	3 (19%)	–	–	–
	Time to ICB (months)	2 (n=1)<br>3 (n=1)<br>21 (n=1)	18 (n=1)<br>19 (n=1)<br>21 (n=1)	–	–	–
Assays	Tumor supernatants	12 (60%)	8 (50%)	–	2 (50%)	–
	Flow cytometry (D0)	17 (85%)	13 (81%)	–	4 (100%)	–
	Flow cytometry (D3)	19 (95%)	10 (63%)	–	4 (100%)	–
	Luminex	19 (95%)	16 (100%)	2 (100%)	4 (100%)	1 (100%)

* The objective response rate was defined according to RECIST v1.1. Ref: Eisenhauer EA, et al., Eur J Cancer. 2009 Jan;45(2):228-47.

Appendix Table S2. List of each tumor sample indicating weight (when available) and protein content by Bradford assay.

Type Tumoral	New ID	Tumor Weight (g)	Total protein concentration in tumor supernatant (µg/ mL)
Lung	L5	2,10	2629,18
Lung	L7	4,20	4205,79
Lung	L8	3,42	6526,31
Lung	L9	1,90	6748,37
Lung	L10	2,41	1765,37
Lung	L12	2,01	2553,68
Lung	L13	1,78	1943,01
Lung	L14	1,02	2464,85
Kidney	K4	6,57	136,15
Kidney	K5	0,74	575,13
Kidney	K7	1,62	2829,03
Kidney	K8	2,50	3872,71
Kidney	K9	5,11	4094,76
Kidney	K10	3,60	985,94
Kidney	K11	4,40	1296,82
Kidney	K15	16,27	13476,75
Kidney	K16	1,91	5926,75
Kidney	K17	6,87	6670,65
Kidney	K18	3,81	7425,65
Kidney	K19	8,55	3106,60
Head & Neck	HN3	0,52	1097,00
Head & Neck	HN5	1,01	1907,49

Appendix Table S3. Consort flow chart of the different assays performed on tumor samples.

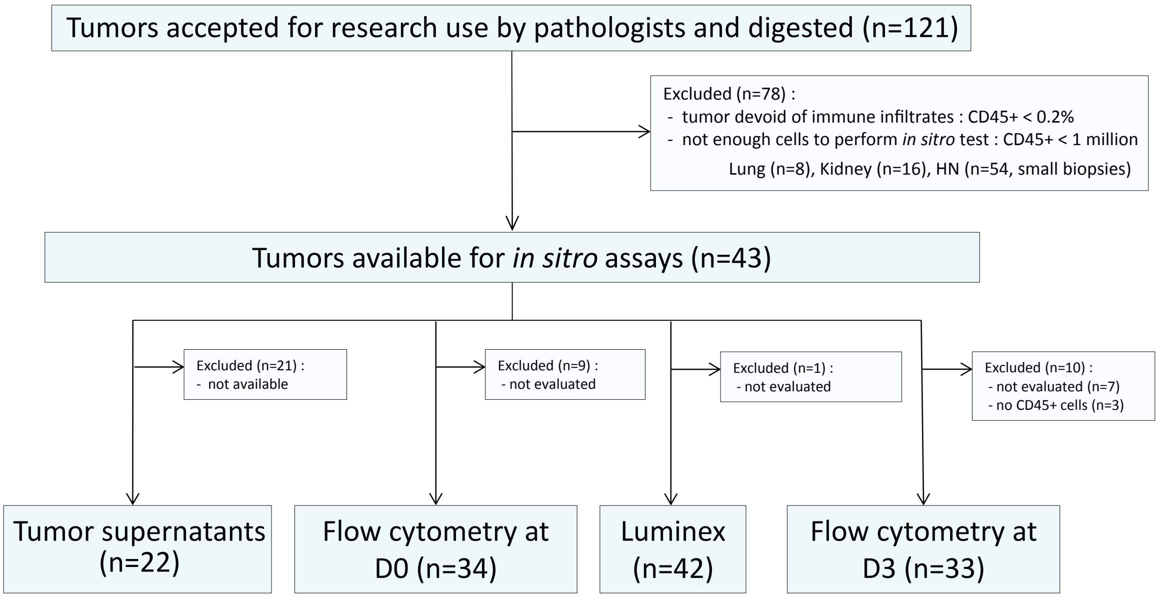

Appendix Table S4. Immune reactivity score (IRS) after anti-PD-1 mAbs stimulation based on Figure 1d algorithm.

Patients	IRS αPD-1	Patients	IRS αPD-1
**L5***	76.47	K7	11.77
**L14***	70.59	L13	11.77
**L16***	58.82	L4	11.77
**L2***	58.82	K10	5.88
**HN3***	52.94	K13	5.88
**L1***	52.94	K21	5.88
**K11***	47.06	K6	5.88
HN4	35.29	B1	0.00
K5	35.29	B2	0.00
L15	35.29	HN5	0.00
L3	35.29	K12	0.00
L6	23.52	K17	0.00
L7	23.52	K19	0.00
K4	17.65	K20	0.00
K9	17.65	K8	0.00
L8	17.65	L11	0.00
HN2	11.77	L12	0.00
K14	11.77	L9	0.00
K15	11.77	O1	0.00
K16	11.77	L10	0.00
K18	11.77	K1	0.00

*IRS > 41.18 are included in blue quadrant

Appendix Table S5. Correspondance between IRS and clinical outcome

SPECIMEN		*In Sitro* RESPONSE (IRS)		CLINICAL RESPONSE	
**Tumor Type**	**Patients ID**	**αPD-1**	**αPD-1 + αCTLA4**	**αPD-1**	**αPD-1 + αCTLA4**
Lung	**L1**	R			
	**L2**	R			
	**L3**	NR		**PD**	
	**L4**	NR			
	**L5**	R			
	**L6**	NR		**PD**	
	**L7**	NR			
	**L8**	NR		**PR**	
	**L9**	NR			
	**L10**	NR			
	**L11**	NR			
	**L12**	NR			
	**L13**	NR			
	**L14**	R			
	**L15**	R			
	**L16**	R			
Kidney	**K1**	NR		**PD**	
	**K2**	NR			
	**K4**	NR			
	**K5**	R			
	**K6**	NR			
	**K7**	NR		**SD**	**PR**
	**K8**	NR			
	**K9**	NR			
	**K10**	NR			
	**K11**	R	R	**SD**	**PR**
	**K12**	NR			
	**K13**	NR			
	**K14**	NR			
	**K15**	NR			
	**K16**	NR			
	**K17**	NR			
	**K18**	NR			
	**K19**	NR			
	**K20**	NR			
	**K21**	NR			
H&N	**HN1**	NR			
	**HN2**	NR			
	**HN3**	R			
	**HN4**	NR			
	**HN5**	NR			
Ovarian	**O1**	NR			
Bladder	**B1**	NR			
	**B2**	NR			

R : responders IRS > 41.2, NR : non responders IRS < 41.2,
PR : partial response, SD : stable disease, PD : progressive disease

AppendixTable S6. Clinical characteristics of the lung cancer patients cohorts for the pronostic value of tumoral CXCL10 mRNA.

Variables		Montreal (n = 51)	Dijon (n = 43)	Total (n = 94)
Gender	Female	27 (53%)	10 (33%)	37 (39%)
	Male	24 (47%)	33 (77%)	57 (61%)
Age, (years)	Median<br>Range	68<br>44-83	66<br>46-85	66<br>44-85
Pathological type, n (%)	Squamous cell carcinoma	6 (12%)	20 (47%)	26 (28%)
	Adenocarcinoma	45 (88%)	22 (51%)	67 (71%)
	Other	0	1 (2%)	1 (1%)
Number of prior lines, n (%)	0	12 (24%)	2 (5%)	14 (15%)
	≥1	39 (76%)	37 (86%)	76 (81%)
	N/A	0	4 (9%)	4 (4%)
Treatment, n (%)	Nivolumab	28 (55%)	42 (98%)	70 (74%)
	Pembrolizumab	17 (33%)	0	17 (18%)
	Other	6 (12%)	0	6 (6%)
	N/A	0	1 (2%)	1 (1%)

Appendix Table S7. Contingency IL-2/anti-PD-1 responders (R) *versus* non-responders (NR)

		**αPD-1**		**Total**
		**R**	**NR**	
**IL-2**	**R**	3	8	11
	**NR**	3	19	22
	**Total**	6	27	33

Appendix Table S8. Scoring of hypo-responsiveness for each tumor sample based on Figure 4d and reversion by αKIR + αPD-1.

Patients	HRS αPD-1	HRS αKIR:αPD-1
**K12***	11.11	0.00
**K14***	11.11	0.00
**L9***	11.11	NA
**B2***	7.41	NA
**K4***	7.41	0.00
**L11***	7.41	NA
HN3	3.70	NA
K1	3.70	NA
K10	3.70	33.33
K11	3.70	18.52
K19	3.70	0.00
K5	3.70	3.70
K6	3.70	NA
K8	3.70	3.70
K9	3.70	7.41
L3	3.70	NA
L5	3.70	NA
B1	0.00	NA
HN2	0.00	NA
HN4	0.00	NA
HN5	0.00	0.00
K13	0.00	NA

Patients	HRS αPD1	HRS αKIR:αPD-1
K15	0.00	0.00
K16	0.00	0.00
K17	0.00	0.00
K18	0.00	0.00
K20	0.00	NA
K21	0.00	NA
K3	0.00	NA
K7	0.00	NA
L1	0.00	NA
L10	0.00	NA
L12	0.00	NA
L13	0.00	NA
L14	0.00	NA
L15	0.00	NA
L16	0.00	NA
L2	0.00	NA
L4	0.00	NA
L6	0.00	NA
L7	0.00	NA
L8	0.00	NA
O1	0.00	NA

*Hypo responding tumors to anti-PD1 mAbs are indicated in violet quadrant
