# Supplementary material for: Immunodynamics of explanted human tumors for immuno‐oncology
Source: EMBO Mol Med. 2020 Dec 29;13(1):e12850. doi: 10.15252/emmm.202012850 (PMC7799366; doi:10.15252/emmm.202012850)
Supplement: Supplementary file 2 — Expanded View Figures PDF [file EMMM-13-e12850-s002.pdf]

Expanded View Figures

Figure EV1. TCR-dependent soluble factors (SF) and correlations with immune effector functions after PD-1 blockade.

A Representative micrograph pictures of TIL densities in stroma surrounding the tumor or tumor nests in two cases containing high (15%, (A)) or low (0.7%, (B)) $CD45^+$ TILs in a surgical specimen. Scale bar representing 100 μm is indicated on the picture.

B Left panel: Heatmap of the non-supervised hierarchical clustering using 27 SFs, segregating patterns of immune reactivity of the TME ($n = 42$) following a 60-h stimulation with anti-CD3 and anti-CD28 mAbs (TCR cross-linking). The heatmap shows *z* score-normalized concentration of soluble factors. Each column represents a tumor and each row a SF. Fold ratio of SF concentrations after TCR cross-linking over the concentrations in untreated cells (medium) were $\log_2$ transformed. Similar data were obtained using isotype control mAbs instead of medium (Appendix Fig S2). The color gradient from purple up to orange indicates increasing gradients of concentrations. Missing values are shown in gray. Both rows and columns are clustered using correlation distance and average linkage. Right panel: distribution of fold ratio of each SF following TCR cross-linking.

C Spearman correlation matrices of SFs and FACS-based effector functions post-PD-1 blockade (fold ratio over medium) for R_{clus} and NR_{clus} tumors according to the clustering of Fig 1B. For each tumor sample, only FACS-based effector functions with data ≥ 500 events in are represented here. *P values < 0.05.

D Assessment of the concordance of the clustering score (R_{clus} and NR_{clus}) and the immune responsive score (IRS_{low} and IRS_{high}; above) and corresponding sensitivity and sensibility values of the IRS for the 42 patients (below). The best cutoff value with the highest likelihood ratio is framed in red.

Source data are available online for this figure.

© 2021 The Authors

A

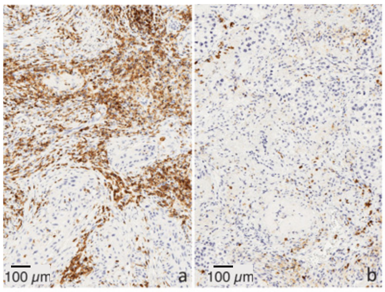

B

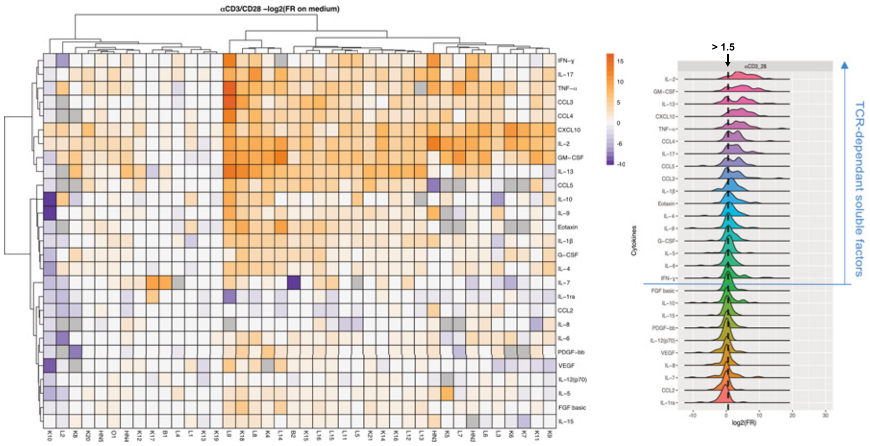

C

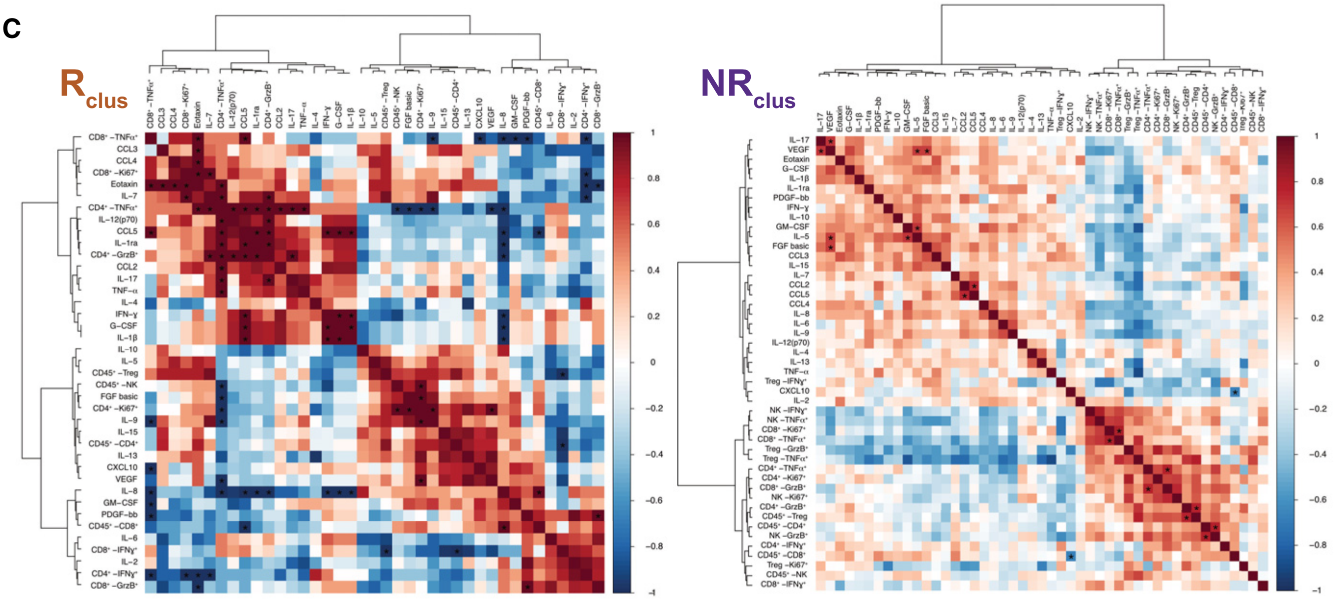

D

	R_{clus}	NR_{clus}	Total
IRS_{high}	6	1	7
IRS_{low}	1	34	35
Total	7	35	42

Cutoff	Sensitivity%	95% CI	Specificity%	95% CI	Likelihood ratio
> 2.941	100	59.04% to 100%	40	23.87% to 57.89%	1.667
> 8.824	100	59.04% to 100%	51.43	33.99% to 68.62%	2.059
> 14.71	100	59.04% to 100%	74.29	56.74% to 87.51%	3.889
> 20.59	100	59.04% to 100%	82.86	66.35% to 93.44%	5.833
> 29.41	100	59.04% to 100%	88.57	73.26% to 96.8%	8.75
> 41.18	85.71	42.13% to 99.64%	97.14	85.08% to 99.93%	30
> 50	71.43	29.04% to 96.33%	97.14	85.08% to 99.93%	25
> 55.88	42.86	9.899% to 81.59%	97.14	85.08% to 99.93%	15
> 64.71	28.57	3.669% to 70.96%	100	90% to 100%	
> 73.53	14.29	0.361% to 57.87%	100	90% to 100%	

Figure EV1.

© 2021 The Authors

Figure EV2. Differential cell surface expressions of activation and exhaustion markers in IRS_{high} vs IRS_{low} tumor samples.

A–G Flow cytometry determination of various cell surface markers (as indicated) within $CD45^-$ cells or different tumor immune subsets among $CD45^+$ cells between the two groups of tumors (IRS_{high} vs IRS_{low}), according to the clustering depicted in Fig 1D. Box plots display group of numerical data through their 3rd and 1st quartiles (box), mean (central band), minimum and maximum (whiskers). In each box plots, each dot represents one tumor. Statistical analyses: Wilcoxon rank-sum test, ns. $P > 0.05$, $*P < 0.05$. Only data ≥ 500 events were plotted here. N/A: data not available or < 500 events.

 © 2021 The Authors

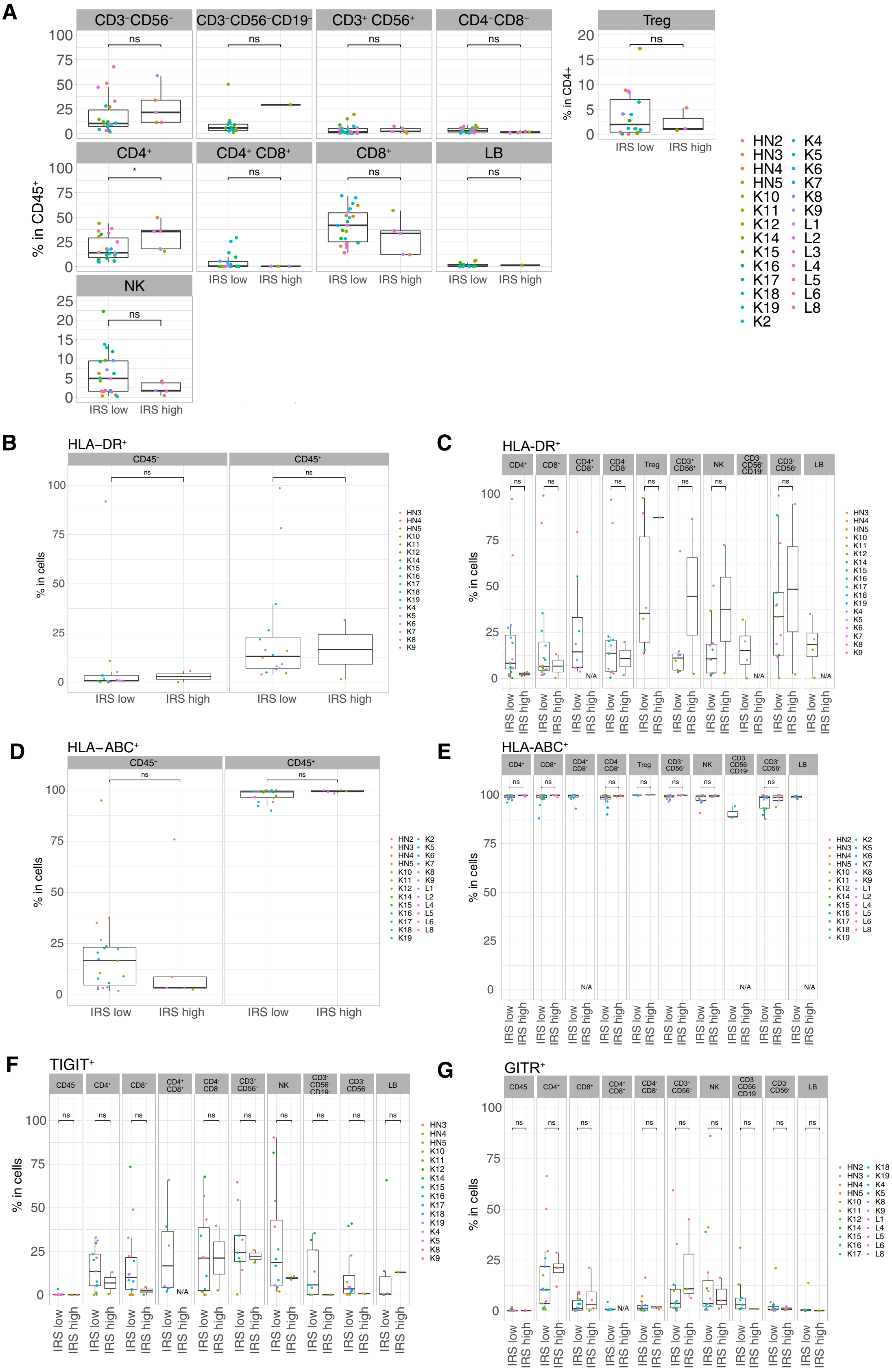
A
CD3⁻CD56⁻
CD3⁻CD56⁻CD19⁻
CD3⁺ CD56⁺
CD4⁻CD8⁻
Treg
CD4⁺
CD4⁺ CD8⁺
CD8⁺
LB
NK
% in CD45⁺
% in CD4+
ns
*
IRS low
IRS high
HN2 HN3 HN4 HN5 K10 K11 K12 K14 K15 K16 K17 K18 K19 K2 K4 K5 K6 K7 K8 K9 L1 L2 L3 L4 L5 L6 L8
B
HLA–DR⁺
CD45⁻
CD45⁺
% in cells
C
HLA-DR⁺
CD4⁺
CD8⁺
Treg
NK
LB
N/A
D
HLA–ABC⁺
E
HLA-ABC⁺
F
TIGIT⁺
G
GITR⁺

Figure EV2.

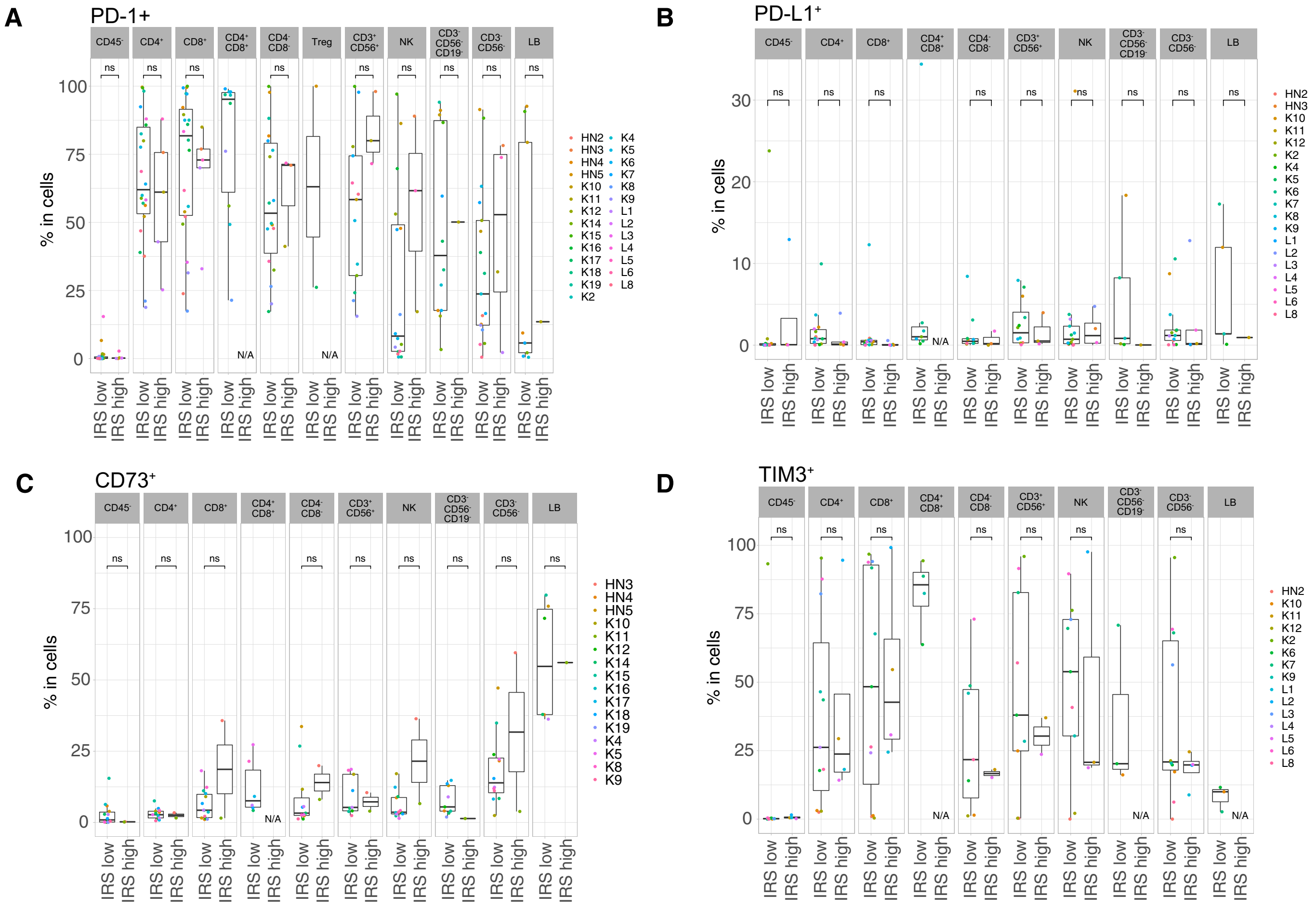

Figure EV3. Differential cell surface expression of PD-L1, PD-1, CD73 and TIM3 in IRS_{high} vs IRS_{low} tumor samples.

A–D Flow cytometry determination of various cell surface markers (as indicated) within different tumor immune subsets or CD45- cells between the two groups of tumors (IRS_{high} vs IRS_{low}), according to the clustering depicted in Fig 1D. Box plots display group of numerical data through their 3rd and 1st quartiles (box), mean (central band), minimum and maximum (whiskers). In each bar graph, each dot represents one tumor. Statistical analyses: Wilcoxon rank-sum test, ns. $P > 0.05$. Only data ≥ 500 events were plotted here. N/A: data not available or < 500 events.

© 2021 The Authors

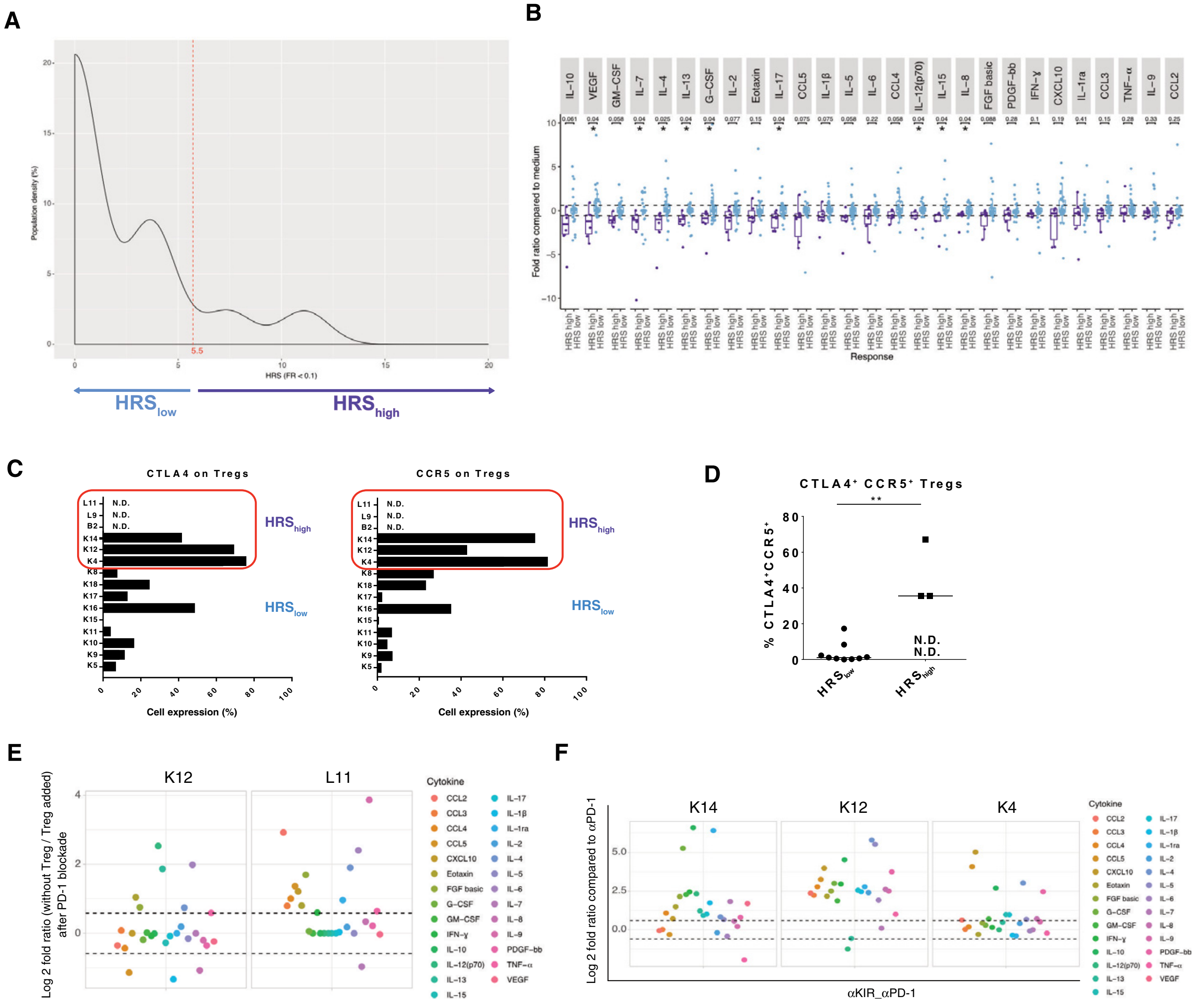

Figure EV4. Hypo-responding tumors with anti-PD-1 mAbs.

A Density of patients for each hypo-responsive score (HRS) of the whole tumor cohort. The HRS was calculated taking into account all the 27 SFs. A tumor was considered "hypo-responding" (or HRS_{high}) when the HRS ≥ 5.5, as underlined in light violet.

B Bar graph according to the fold ratio range between HRS_{high} (> 5.5) and HRS_{low} (< 5.5). Box plots display group of numerical data through their 3rd and 1st quartiles (box), mean (central band), minimum and maximum (whiskers). Each dot represents one tumor for each SF. Statistical analyses: Wilcoxon rank-sum test and *P* values with a Benjamini–Hochberg (BH) correction procedure ($^*P < 0.05$) are indicated for each SF to interpret the significance between HRS_{high} vs HRS_{low} groups of tumors.

C Detailed CTLA4 and CCR5 membrane expression levels (percentage) on each of the 12 (available) tumors evaluated at RT. N.D.: measure could not be performed.

D Flow cytometric dot plot analysis of the percentages of Treg co-expressing CCR5 and CTLA4 on their surface expression in the immune infiltrate after dissociation in 12 (available) cases. Black line represents the mean of the group. Statistical analyses: Wilcoxon rank-sum test, $^{**}P < 0.01$. N.D.: not done.

E *In sitro* assay with or without Treg. Same experimental setting was performed as the one described in Fig 1A after cell sorting of $CD25^+PD\text{-}1^+$ Treg from TILS, alone (without Treg), or restored with Treg cells at a 10:1 ratio E:Treg ratio (Treg added), then stimulated with anti-PD-1 mAbs. Upper and lower dashed line represent a 1.5-fold increase and decrease, respectively, in soluble factor release.

F Efficacy of anti-KIR mAbs in preventing hypo-responsiveness to PD-1 blockade for K14, K12 and K4. All soluble factors (SF), i.e., HRS components, in anti-PD-1 + anti-KIR mAbs were compared to anti-PD-1 mAbs. Upper and lower dashed line represent a 1.5-fold increase and decrease, respectively, in soluble factor release.

Source data are available online for this figure.
