# Supplementary material for: Immunodynamics of explanted human tumors for immuno‐oncology
Source: EMBO Mol Med. 2020 Dec 29;13(1):e12850. doi: 10.15252/emmm.202012850 (PMC7799366; doi:10.15252/emmm.202012850)
Supplement: Supplementary file 4 — Source Data for Expanded View [file EMMM-13-e12850-s005.pdf]

Figure EV1 Source Data

L9

CD45+ evaluated by FACS = 15%

CD45+ evaluated by IHC = 80%, CD45 stroma >800/mm2

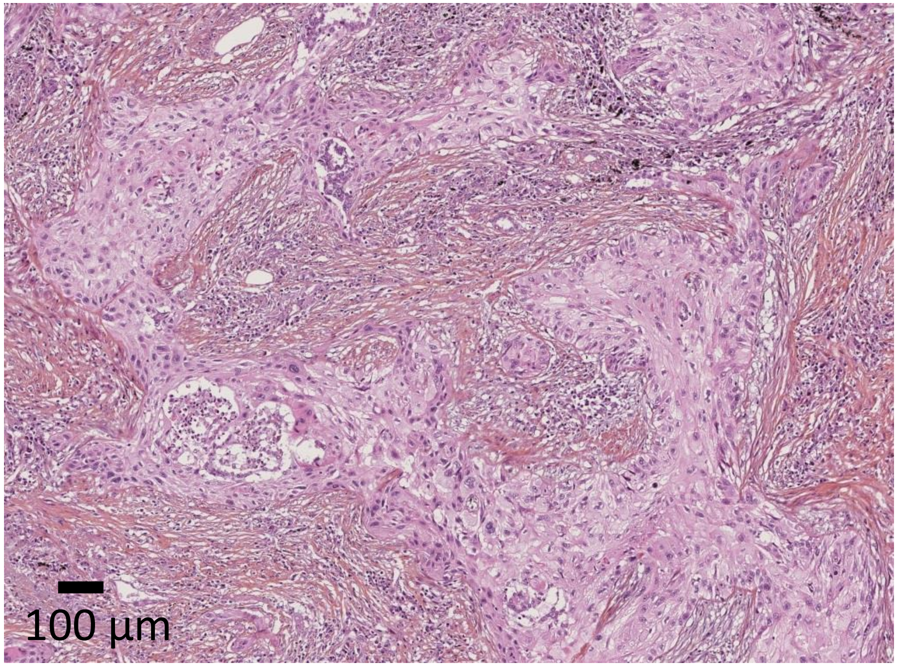

hematoxylin-eosin

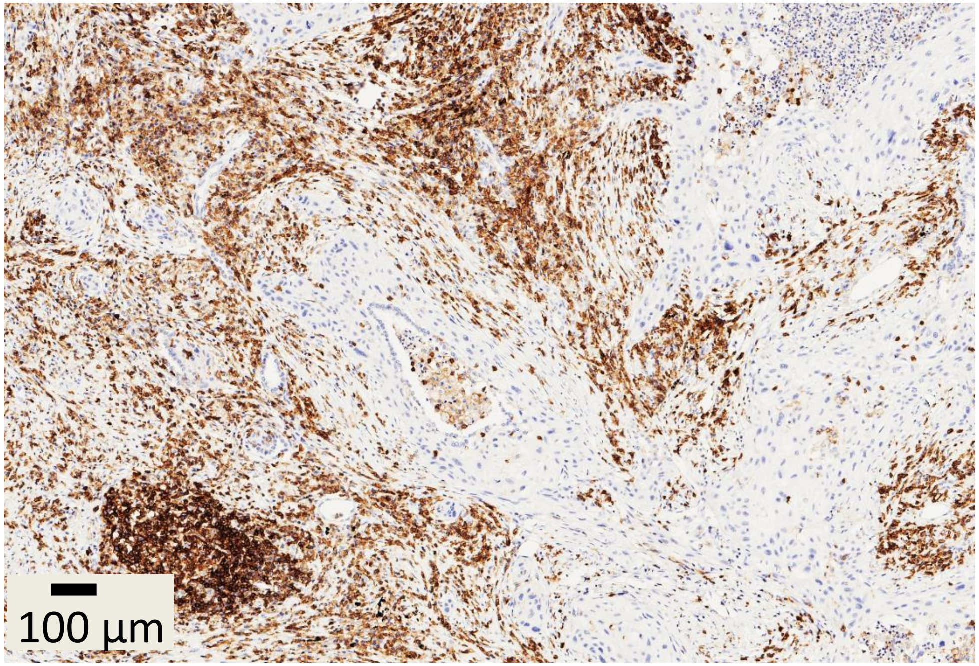

CD45 immunodetection
marron color = CD45+ cells

L14

CD45+ evaluated by FACS = 4,5%

CD45+ evaluated by IHC = 80%, CD45 stroma >1000/mm2

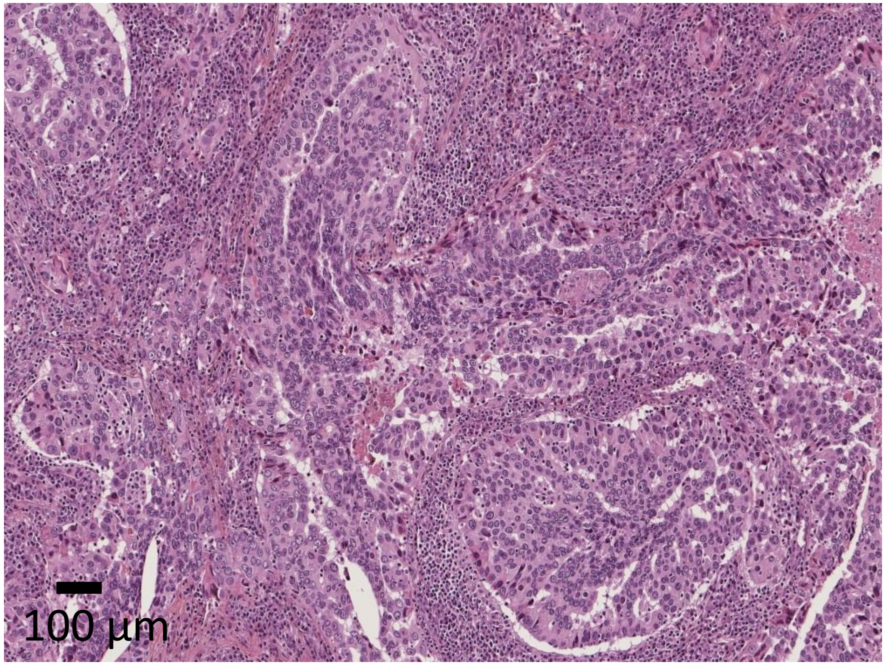

hematoxylin-eosin

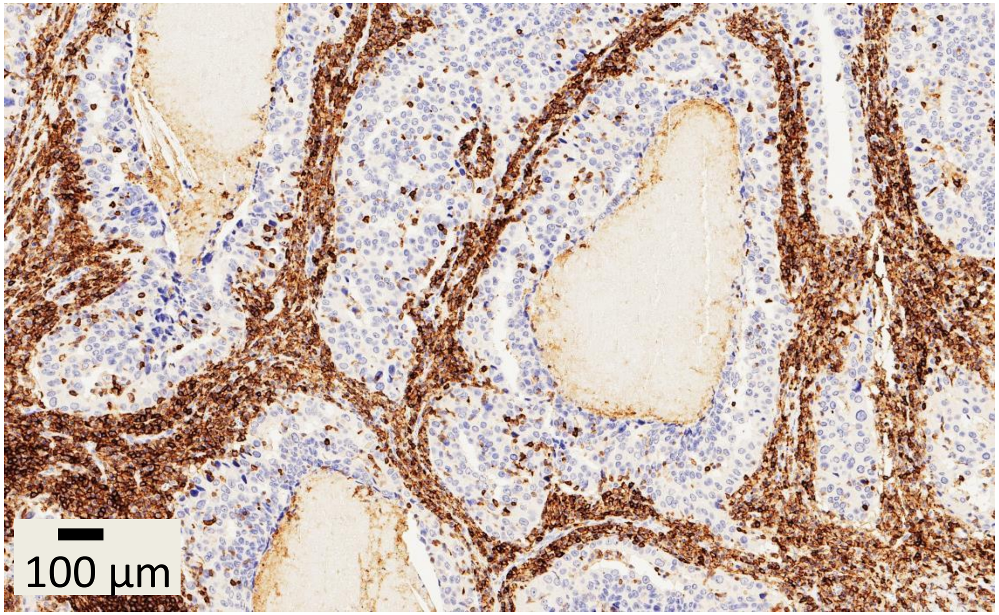

CD45 immunodetection
marron color = CD45+ cells

K14

CD45+ evaluated by FACS = 1,2%

CD45+ evaluated by IHC = 20%, CD45 stroma = 100-150 mm2

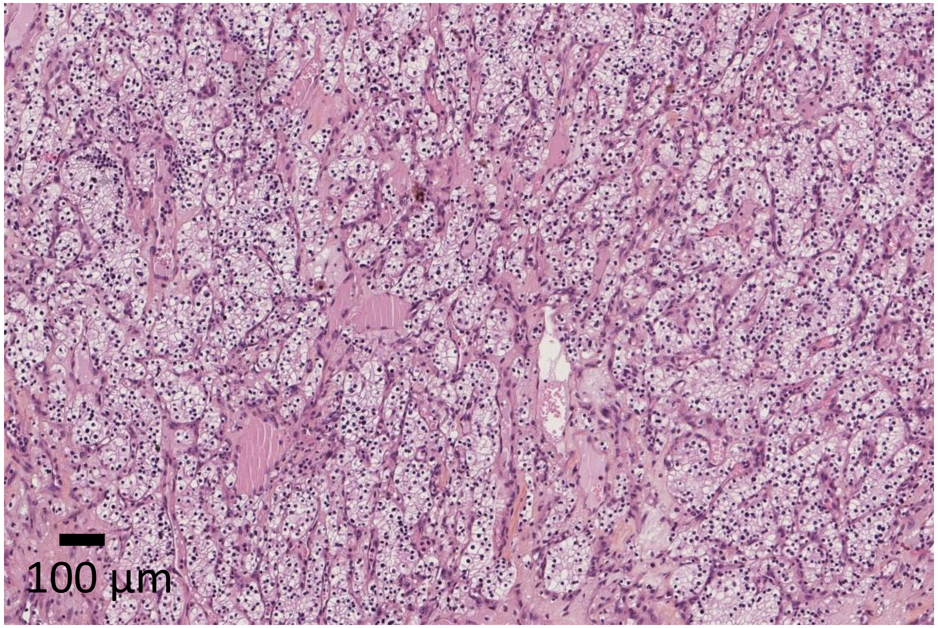

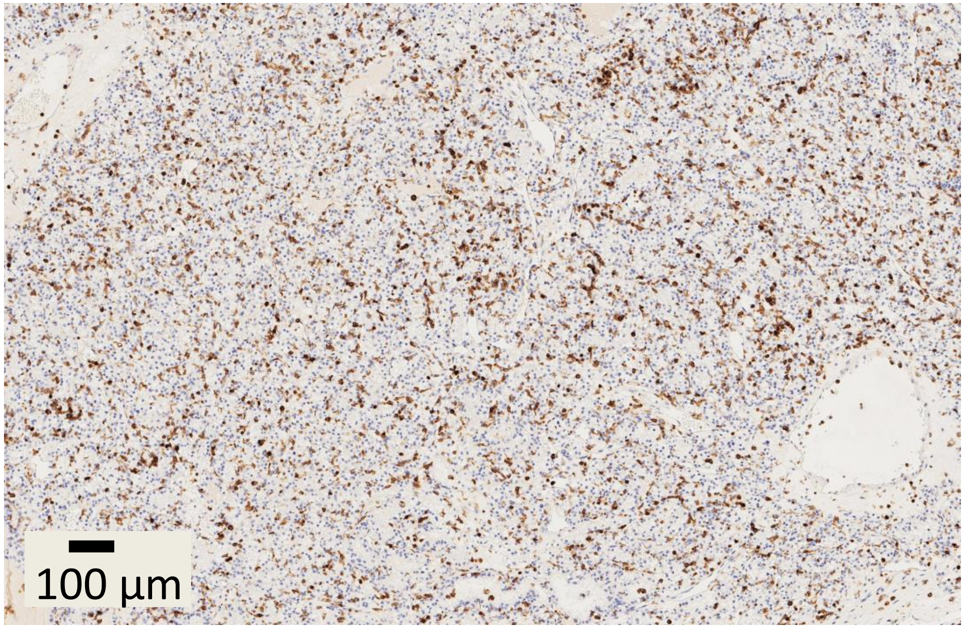

hematoxylin-eosin

CD45 immunodetection
marron color = CD45+ cells

K15

CD45+ evaluated by FACS = 0,6%
CD45+ evaluated by IHC = 20%, CD45 stroma = 20-100/mm2

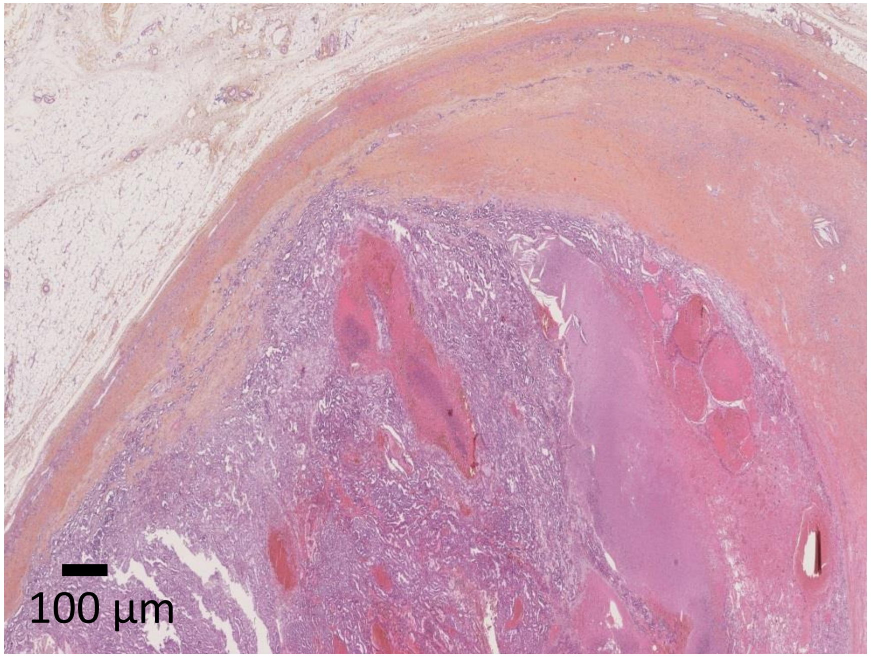

hematoxylin-eosin

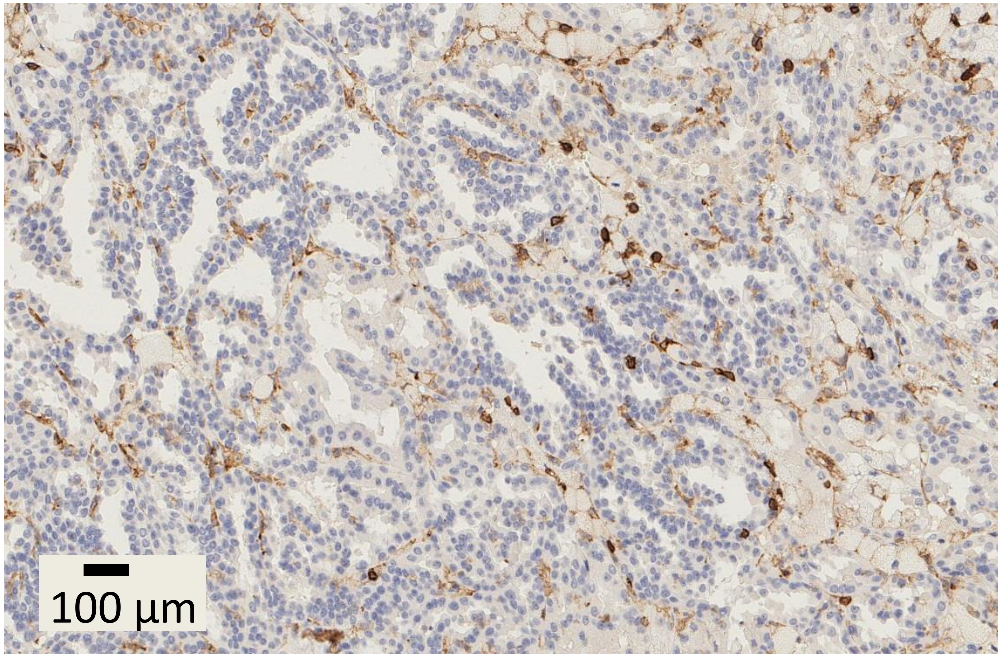

CD45 immunodetection
marron color = CD45+ cells

L10

CD45+ evaluated by FACS = 0,7%

CD45+ evaluated by IHC < 10%, CD45 stroma : rare diffuse

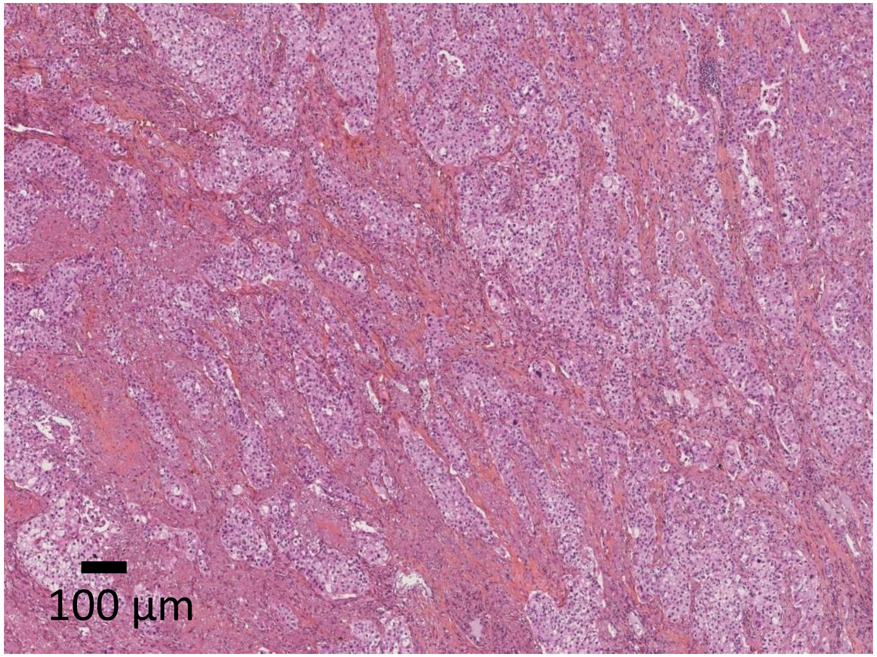

hematoxylin-eosin

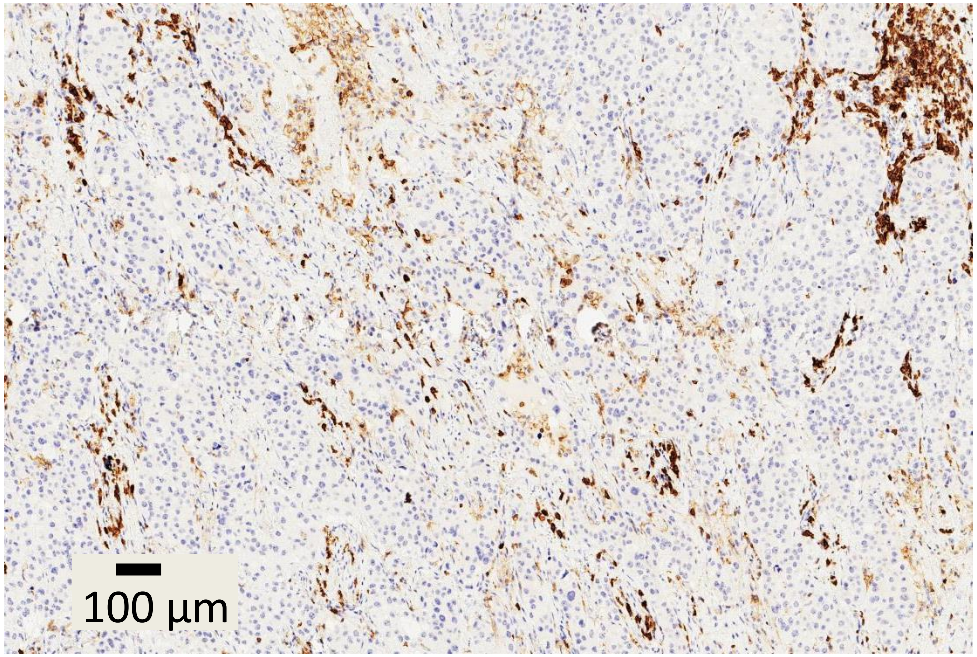

CD45 immunodetection
marron color = CD45+ cells

CD45+ < 0,2%

CD45+ evaluated by FACS = 0,2%
CD45+ evaluated by IHC = 10%, CD45 stroma : 50/mm2

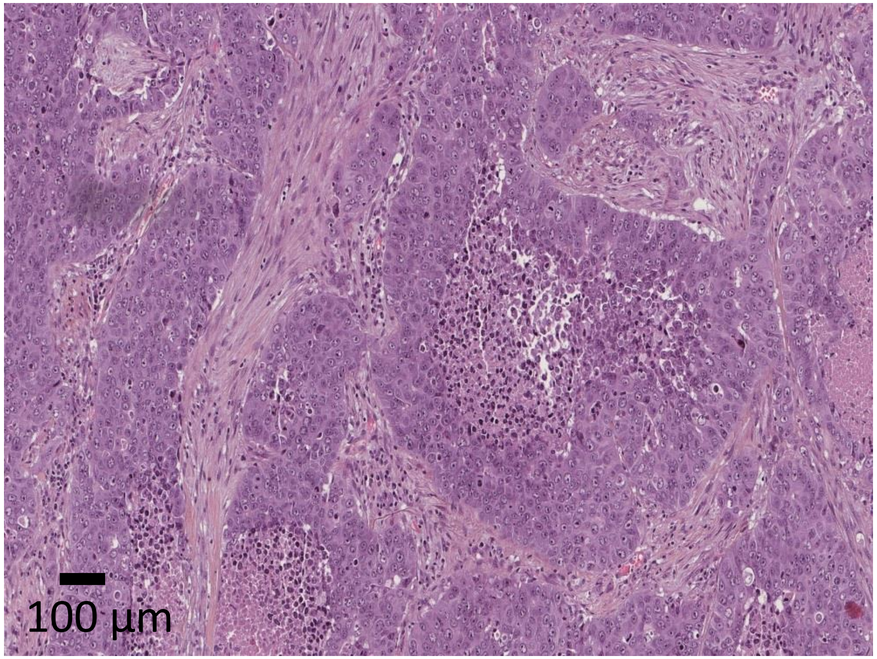

hematoxylin-eosin

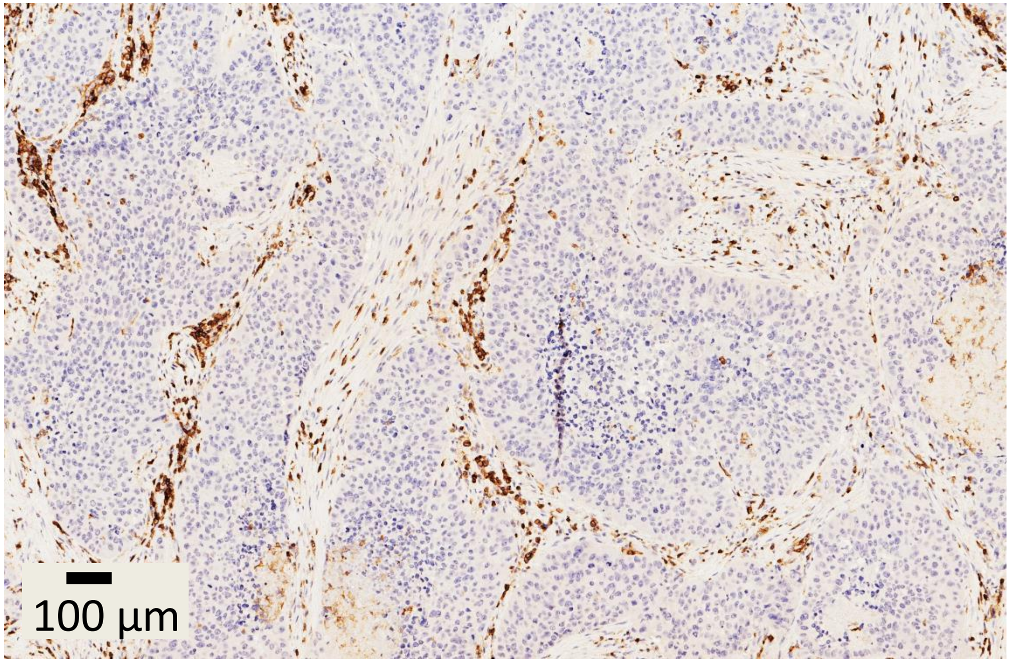

CD45 immunodetection
marron color = CD45+ cells

Figure EV4

	Tregs	CCR5+	CTLA4+
Figure EV4C	K5	1,23	6,32
	K9	6,51	11,01
	K10	4,06	15,99
	K11	6,26	3,6
	K16	34,57	48,15
	K15	0,00	6,52
	K17	1,6	12,5
	K18	22,5	24,17
	K8	26,26	7,07
	K4	80,82	75,34
	K12	42,22	68,89
	K14	74,73	41,28
Figure EV4D	CTLA4+CCR5+ Tregs	HRS low	HRS high
	K5	0,2	
	K8	2,28	
	K10	1,09	
	K17	1,35	
	K15	0,01	
	K9	0,63	
	K12		35,56
	K4		67,12
	K14		35,59
	K16	17,28	
	K11	0,53	
	K18	8,33	
